# Mechanisms, clinical manifestations and management of cardiovascular diseases in ANCA-associated vasculitis

**DOI:** 10.1093/rheumatology/keag268

**Published:** 2026-07-02

**Authors:** Emanuele Chiara, Giacomo Bagni, Alessandro Raho, Filippo Fagni, Aladdin J Mohammad, Giacomo Emmi

**Affiliations:** Department of Medical, Surgery and Health Sciences, University of Trieste, Trieste, Italy; Clinical Medicine and Rheumatology Unit, Cattinara University Hospital, Trieste, Italy; Department of Clinical and Biological Sciences, University of Turin, Turin, Italy; Department of Medical, Surgery and Health Sciences, University of Trieste, Trieste, Italy; Clinical Medicine and Rheumatology Unit, Cattinara University Hospital, Trieste, Italy; Department of Medicine 3—Rheumatology and Immunology, Friedrich-Alexander-Universität (FAU) Erlangen-Nürnberg and Universitaetsklinikum Erlangen, Erlangen, Germany; Section of Rheumatology, Department of Clinical Sciences, Lund University, Lund, Sweden; Department of Medicine, University of Cambridge, Cambridge, UK; Department of Medical, Surgery and Health Sciences, University of Trieste, Trieste, Italy; Clinical Medicine and Rheumatology Unit, Cattinara University Hospital, Trieste, Italy; Department of Medicine, Monash Medical Centre, Centre for Inflammatory Diseases, Monash University, Melbourne, Australia

**Keywords:** ANCA-associated vasculitis, EGPA, eosinophils, cardiovascular disease, thrombosis, atherosclerosis, acute myocardial infarction, ischaemic stroke, venous thromboembolism

## Abstract

ANCA-associated vasculitides (AAVs) are rare diseases characterized by small-vessel necrotizing vasculitis, multiorgan involvement and positivity for ANCAs. The main phenotypes are granulomatosis with polyangiitis, microscopic polyangiitis and eosinophilic granulomatosis with polyangiitis, representing distinct yet partially overlapping entities in terms of pathogenesis, clinical expression and therapeutic management. Patients with AAV face a substantial cardiovascular (CV) and thrombotic risk, with higher rates of myocardial infarction, ischaemic stroke and venous thromboembolism than the general population. The excess CV burden reflects a complex, time-dependent interplay between disease-related inflammation, traditional CV risk factors and treatment-related toxicity, with inflammatory activity emerging as a key driver of early CV events. Across the disease course, this evolving risk profile requires multidisciplinary management to limit CV damage accrual and related mortality. This review integrates evidence on pathogenesis, clinical manifestations and management of CV disease in AAV, highlighting its time-dependent trajectory and key unmet needs.

Rheumatology key messages

- Cardiovascular risk in AAV is high vs the general population, bimodal, inflammation- and toxicity-driven.
- Arterial, venous and cardiac manifestations contribute to cardiovascular morbidity and mortality in AAV.
- Optimal cardiovascular management in AAV requires integrated immunosuppressive and preventive strategies.

## Introduction

ANCA-associated vasculitides (AAVs) are primary systemic diseases characterized by necrotizing inflammation predominantly affecting small blood vessels. The main clinical entities are granulomatosis with polyangiitis (GPA), microscopic polyangiitis (MPA) and eosinophilic granulomatosis with polyangiitis (EGPA). GPA and MPA are commonly associated with PR3- and MPO-ANCA, respectively, whereas ∼30% of patients with EGPA are ANCA-positive, mainly MPO [1]. AAV frequently involve the ear–nose–throat region, lungs, kidneys, eyes, skin and peripheral nervous system, although virtually any organ may be affected.

Current management relies on remission induction with glucocorticoids combined with immunosuppressive agents, followed by maintenance therapy to reduce relapse risk [2]. These strategies have markedly improved early survival; however, long-term outcomes remain suboptimal, with substantial chronic damage and comorbidities [3] and an overall mortality ∼2.7-fold higher than in the general population [4].

Cardiovascular (CV) disease has emerged as a major determinant of long-term prognosis and a leading cause of death in AAV [5–7]. Population-based studies show a significantly increased risk of CV events compared with matched controls, even after adjustment for traditional CV risk factors and prior CV disease, with up to a threefold excess risk [8–11]. This burden includes major arterial and venous manifestations such as myocardial infarction, ischaemic stroke and venous thromboembolism.

The increased CV risk in AAV is not fully explained by traditional CV risk factors. Although disease onset typically occurs between the fifth and seventh decades of life, accumulating evidence supports an independent role of systemic inflammation. A large population-based study across autoimmune diseases showed that CV risk is disproportionately higher in younger patients and only partially accounted for by classical risk factors, implicating inflammation as a driver of premature CV disease [12]. In AAV, inflammation and vascular injury likely contribute substantially to early arterial and venous events.

The temporal pattern of CV disease reinforces this concept. CV risk peaks early, paralleling inflammatory activity, but remains elevated during remission, reflecting irreversible vascular damage, residual inflammation, traditional risk factors and treatment-related toxicity. While immunosuppressive therapies are essential to limit early vascular injury, long-term exposure, particularly to glucocorticoids, contributes to cumulative CV risk. Conventional preventive strategies may mitigate residual risk, although disease-specific evidence in AAV remains limited.

Taken together, these considerations highlight the need for a comprehensive approach to CV disease in AAV. This review summarizes current pathophysiological concepts and discusses implications for CV risk stratification, monitoring and therapeutic intervention in clinical practice.

## Mechanisms of CV damage in AAV: the role of inflammation

### Common inflammatory and vascular mechanisms in AAV

CV disease in AAV is closely related to systemic, vascular inflammation and direct cardiac inflammatory involvement (e.g. myocarditis and pericarditis). Experimental and clinical models consistently demonstrate that CV damage arises from multiple immune-mediated mechanisms involving innate immunity (neutrophils, eosinophils, monocytes and macrophages), adaptive immunity (T and B lymphocytes), complement activation and autoantibodies, including ANCA. While several pathogenic pathways are shared across AAV subtypes, particularly GPA and MPA, relevant differences in mechanisms and organ involvement can be identified [13, 14].

A common pathogenic framework underlying CV involvement in all AAV is represented by the combination of endothelial dysfunction, accelerated atherosclerosis and a prothrombotic diathesis. Endothelial injury is primarily driven by reactive oxygen species (ROS)-mediated vascular damage induced by neutrophils, especially in GPA and MPA, following their activation through the interaction between circulating ANCAs and surface-expressed myeloperoxidase (MPO) and proteinase 3 (PR3) [15]. Activated neutrophils exhibit enhanced adhesion to the vascular wall through increased expression of intracellular adhesion molecules (ICAMs) and selectins, a process promoted by pro-inflammatory cytokines such as TNF and IL-1β or triggered by local stimuli, including bacterial lipopolysaccharides [16].

Neutrophil-rich vascular infiltration represents a hallmark of AAV and promotes granuloma formation through interactions with resident monocytes, the release of neutrophil extracellular traps (NETs) [13] and activation of Th1 and Th17 lymphocytes, particularly in granulomatous phenotypes such as GPA and EGPA [17]. Activated neutrophils release large amounts of granular proteinases, potent generators of ROS, leading to local vascular damage [18]. These mechanisms further enhance the surface expression of MPO and PR3 on intravascular neutrophils, contributing to loss of immune tolerance and perpetuation of ANCA-mediated inflammation [19]. Binding of ANCAs to these antigens sustains a self-amplifying cycle of neutrophil activation, vasculitic injury and ROS generation. Complement activation, particularly through the C5a fraction released by activated neutrophils and enhanced by TNF, further contributes to endothelial damage [20].

CRP may further link inflammation, complement activation and atherosclerosis [21]. In AAV, ANCA-induced (especially anti-MPO) NET formation promotes platelet activation and generation of monomeric CRP, amplifying thrombogenesis and vascular inflammation through classical and alternative complement pathways activation [22, 23].

### Inflammation-driven atherosclerosis and thrombosis in AAV

The inflammatory mechanisms described above contribute directly to accelerated atherosclerosis, a major determinant of arterial ischaemic events in AAV. Direct vascular inflammation, increased leucocyte adhesion, endothelial activation and complement engagement promote intimal hyperplasia and atherosclerotic plaque formation [24, 25]. In addition, systemic inflammation characterized by elevated levels of IL-1β, IL-6, TNF and CRP enhances endothelial dysfunction and drives immune-mediated vascular remodelling, ultimately leading to vascular wall thickening and plaque progression [24, 26].

Beyond atherosclerosis, ANCAs and neutrophil activation interfere with coagulation pathways, favouring a prothrombotic state that affects both arterial and venous circulations. In a prospective study, patients with active AAV showed elevated circulating thrombin–antithrombin complexes, D-dimers and coagulation factors bound to their natural inhibitors within the intrinsic coagulation pathway [27]. These markers of hypercoagulability correlated with disease activity, ANCA titre, CRP levels and proteinuria, reinforcing the link between inflammation and thrombosis [27]. Consistently, von Willebrand factor antigen levels are increased in childhood-onset AAV and correlate with disease activity, further reflecting endothelial activation and thrombotic risk [28].

The strong association between inflammatory burden and CV risk is supported by the clustering of arterial and venous events during early disease phases, when systemic inflammation is most pronounced. However, a procoagulant state persists even during clinical remission [29, 30]. In this context, MPO-ANCA itself may exert direct pro-atherogenic effects, contributing to sustained CV risk independently of overt disease activity [31]. These findings underscore that inflammation-driven thrombosis in AAV is not confined to active disease but may represent a long-lasting pathogenic feature.

### Mechanisms of cardiac involvement in AAV

Cardiac involvement in AAV may be either ischaemic or inflammatory. In addition to classic plaque rupture, myocardial ischaemia can result from coronary vasospasm, small-vessel vasculitis or myocardial infarction with non-obstructive coronary arteries (MINOCA) [32, 33]. The inflammatory pathways described above also directly contribute to myocardial damage. ANCA-mediated neutrophil activation, NETosis, excessive ROS production and complement activation can injure cardiomyocytes, leading to fibrosis, ventricular dysfunction, heart failure and arrhythmias. Fibrogenic cytokines, including IL-1, further amplify myocardial remodelling. Pericardial involvement and valvular damage have also been reported.

### EGPA and CV damage: the role of eosinophils

In EGPA, neutrophils remain relevant contributors to vascular damage, but eosinophils provide an additional and distinct prothrombotic and cardiotoxic stimulus, partly independent of neutrophil-mediated mechanisms [34]. Eosinophils share with neutrophils the capacity to generate ROS and release granule proteins but possess unique intrinsic procoagulant properties [35]. Activated eosinophils release granules rich in NADPH-oxidase and eosinophil peroxidase, which have a particularly high oxidative potential. Eosinophil-specific granule proteins, including eosinophilic cationic protein (ECP) and major basic protein (MBP), can directly interfere with the coagulation cascade, mainly through factor XII-dependent pathways [36].

Additional prothrombotic mechanisms in EGPA include eosinophil extracellular trap formation (EETosis), which has been implicated in atherothrombosis similarly to NETosis [37], initiation of coagulation through tissue factor releases during eosinophil degranulation, inhibition of endothelial thrombomodulin by MBP and platelet activation by both MBP and eosinophil peroxidase [38]. Activated intravascular eosinophils also expose a procoagulant oxidized aminophospholipid scaffold that supports tissue factor expression, facilitating thrombin generation and platelet adhesion [39]. Moreover, fibrinolysis appears impaired in EGPA, with fibrin clots showing reduced permeability and increased resistance to lysis compared with those of healthy individuals [39].

In EGPA, direct eosinophilic infiltration of myocardial tissue and eosinophil degranulation induce cellular injury through ROS generation, ECP and MBP release and EETosis, ultimately leading to cardiomyocyte death [40, 41]. Eosinophils may further promote myocardial fibrosis via ECP-mediated collagen contraction and secretion of profibrotic cytokines such as transforming growth factor-β and IL-1β [38].

**Figure 1:**
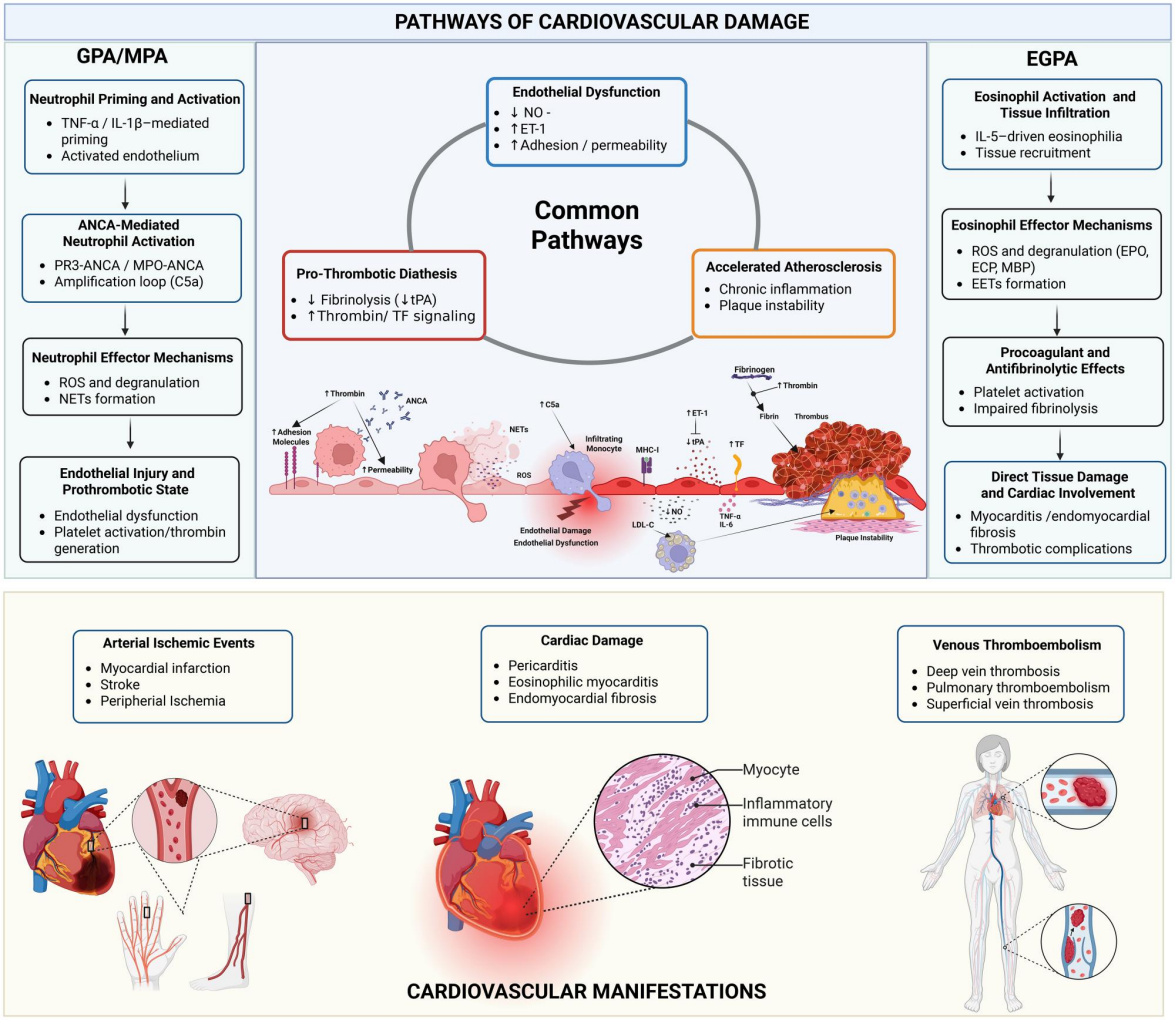
Pathways of cardiovascular damage in ANCA-associated vasculitis. The figure illustrates shared and disease-specific mechanisms contributing to cardiovascular involvement in ANCA-associated vasculitis. In granulomatosis with polyangiitis (GPA) and microscopic polyangiitis (MPA), ANCA-mediated neutrophil activation promotes endothelial dysfunction, oxidative stress, complement activation and prothrombotic pathways, leading to accelerated atherosclerosis and arterial and venous events. In eosinophilic granulomatosis with polyangiitis (EGPA), eosinophil activation and degranulation provide additional prothrombotic and profibrotic stimuli, contributing to vascular and cardiac damage. These inflammatory and immune-mediated pathways converge into a spectrum of cardiovascular manifestations, including arterial ischaemic events, venous thromboembolism and direct cardiac involvement. ROS, reactive oxygen species; NETs, neutrophil extracellular traps; EPO, eosinophil peroxidase; MBP, major basic protein; ECP, eosinophil cationic protein; TF, tissue factor; NO, nitric oxide; ET-1, endothelin-1, MPO-ANCA, myeloperoxidase ANCAs; PR3-ANCA, proteinase 3 ANCAs; EETs, eosinophil extracellular traps; MHC-I, major histocompatibility complex class I; C5a, complement component 5a

Figure 1 summarizes the main pathogenetic pathways of CV damage in AAV.

## CV involvement in ANCA-associated vasculitis

CV involvement in AAV encompasses a broad and heterogeneous spectrum of manifestations, including arterial ischaemic events, venous thromboembolism (VTE) and cardiac disease. These manifestations frequently coexist and may overlap during the disease course, reflecting shared inflammatory, vascular and immune-mediated mechanisms.

Patients with AAV have been reported to show a nearly 30-fold increased risk of deep vein thrombosis (DVT) and a 10-fold increased risk of pulmonary embolism compared with the Swedish general population [9]. In the same population-based AAV cohort, VTE reportedly affected 18% of patients with higher incidence rate early in the disease course [9].

In a meta-analysis of ∼14 000 patients with AAV, Houben *et al.* reported significantly increased risks compared with the general population for total CV events (relative risk, RR 1.65), ischaemic heart disease (RR 1.60) and cerebrovascular events (RR 1.20) [31]. Real-world data from a population-based cohort in southern Sweden further illustrate the cumulative CV burden: after AAV diagnosis, myocardial infarction occurred in 11% of patients [8], stroke in 8% [8, 10]. When stratified by phenotype, myocardial infarction and stroke were observed in 9% and 9% of patients with GPA and in 15% and 6% of those with MPA, respectively [8, 10]. Owing to predominant small-vessel involvement, multiple arterial territories may be affected, and lacunar infarcts and intracerebral haemorrhages are relatively frequent [42].

Below, CV involvement is discussed separately for GPA, MPA and EGPA, integrating arterial, venous and cardiac manifestations within each phenotype. Cumulative frequencies of major events are summarized in Table 1.

**Table 1:** Prevalence (%) of cardiovascular manifestations in AAV subtypes.

		Prevalence (%)		
	Manifestation	GPA (%)	MPA (%)	EGPA (%)
Arterial ischaemic events	Ischaemic stroke	3.6	7.6	1.7
	Myocardial infarction	4.6	9.1	4.0
	Peripheral ischaemia	NR	7.1	NR
Venous thromboembolism	Deep vein thrombosis	3.0	NR	3.3
	Pulmonary embolism	1.3	NR	0.9
Cardiac involvement	Pericarditis	1.2	10.6	26.2
	Myocarditis	NR	NR	12.6
	Cardiomyopathy/heart failure	1.0	17.6	19.4
	Valvular disease	0.2	NR	15.5
	Severe conduction disorder	0.2	NR	NR

Prevalence refers to the cumulative proportion of patients who experienced the specified manifestation during follow-up (or at the defined time-point) in the original source cohort. Estimates were derived exclusively from large, well-characterized cohort or population-based studies reporting explicit numerators and denominators for each AAV subtype. ‘NR’ (not reported) indicates absence of robust diagnosis-specific prevalence data in the primary literature. Incidence rates and mixed-AAV data were not converted into crude proportions to preserve internal consistency.

Follow-up time and timing of assessment varied across the original source studies, ranging from baseline/diagnosis-based evaluations to cumulative estimates over longitudinal follow-up; therefore, prevalence values should be interpreted as source-specific frequencies rather than directly comparable time-standardized measures.

Although derived from heterogeneous cohorts, these data highlight clinically relevant differences across subtypes, particularly the high burden of venous thromboembolism in GPA, heart failure in MPA and direct cardiac involvement in EGPA. Several of these manifestations, especially myocardial infarction, heart failure and cardiac involvement detected by imaging, have been associated with increased long-term mortality in AAV cohorts.

### Granulomatosis with polyangiitis

In a population-based incident cohort of GPA (*n* = 504), cumulative proportions of myocardial infarction and ischaemic stroke during follow-up were 4.6% and 3.6%, respectively [43]. Peripheral arterial ischaemia is less consistently reported as a distinct end point and is often described within broader categories such as cutaneous vasculitis, acral necrosis or limb ischaemia [3, 44].

VTE represents a prominent feature of CV involvement in GPA. An early report by Merkel *et al.* described an incidence of 8.9%, corresponding to 7.0 events per 100 person-years, with a clear association with active disease [45]. Data from the Danish National Hospital Register showed markedly increased incidence rate ratios for pulmonary embolism and DVT within the first 2 years after diagnosis [46]. In a population-based incident cohort, cumulative proportions of DVT and pulmonary embolism were 3.0% and 1.3%, respectively [47]. These findings align with broader AAV cohorts showing clustering of venous events early after diagnosis and during periods of high inflammatory activity, with DVT occurring more frequently than isolated pulmonary embolism [44].

In the Swedish registry-based AAV studies by Borgas *et al.* [8], Tabakovic *et al.* [10] and Liapi *et al.* [9], the myocardial infarction incidence rate in GPA was 1.1 per 100 person-years (95% CI 0.7–1.9) [8]. In Tabakovic *et al.*, the overall AAV stroke incidence rate was 11.3 per 1000 person-years (95% CI 6.9–15.8) and the standardized incidence ratio for stroke in GPA vs the general population was 1.41 (95% CI 0.77–2.37) [10]. In Liapi *et al.*, VTE risk vs the general population was reported separately for DVT and pulmonary embolism in GPA, with standardized incidence ratios of 43.0 (95% CI 19.6–66.4) for DVT and 11.0 (95% CI 3.8–18.2) for pulmonary embolism [9].

Clinically recognized cardiac involvement in GPA appears less frequent but likely underestimated. In the Vasculitis Clinical Research Consortium cohort, cardiac manifestations attributed to GPA were reported in 3.3% of patients, including pericarditis, cardiomyopathy, coronary artery disease, valvular abnormalities and conduction disorders [48]. Imaging-based studies suggest that subclinical cardiac abnormalities are more prevalent and carry prognostic relevance [14, 49].

### Microscopic polyangiitis

Data on arterial events in MPA indicate that ischaemic manifestations may already be present at diagnosis. In an early cohort, digital ischaemia and myocardial infarction were observed in 7.1% and 2.4% of patients, respectively [50]. These events likely reflect the combined impact of vasculitis-related endothelial injury, chronic inflammation, renal involvement and traditional CV risk factors.

Most MPA cohorts report VTE as a composite outcome, limiting precise phenotype-specific estimates. However, mixed-AAV cohorts indicate a predominance of DVT over pulmonary embolism, supporting a venous pattern similar to that observed in GPA and linked to disease activity and procoagulant changes [31, 51].

According to recent Swedish cohort studies, the myocardial infarction incidence rate in MPA was 2.6 per 100 person-years (95% CI 1.7–4.0) [8]. The overall AAV stroke incidence rate was 11.3 per 1000 person-years (95% CI 6.9–15.8) [10], and the standardized incidence ratio for stroke in MPA vs the general population was 2.18 (95% CI 1.24–3.54) [10]. For VTE, the standardized incidence ratio for DVT in MPA was 25.3 (95% CI 8.8–41.8), and the standardized incidence ratio for pulmonary embolism in MPA was 10.3 (95% CI 3.6–17.1) [9].

Cardiac involvement in MPA is more frequently reported than in GPA. In the French Vasculitis Study Group (FVSG) cohort, cardiac failure and pericarditis were present in 17.6% and 10.6% of patients at baseline, whereas myocardial infarction occurred in 2.4% [50]. These manifestations typically occur in the context of severe multisystem disease and significant renal involvement.

### Eosinophilic granulomatosis with polyangiitis

EGPA displays a distinctive CV phenotype, characterized by a high burden of both thrombotic and cardiac manifestations. In the Italian EGPA Consortium cohort, acute arterial and venous thromboembolic events occurred in 22.5% of patients, clustering around the time of diagnosis [52]. Post-diagnosis cumulative proportions for myocardial infarction and ischaemic stroke were 4.0% and 1.7%, respectively. The overall risk of thromboembolic events was significantly higher than in the general population and comparable to that observed in GPA and MPA, with eosinophil-driven vascular injury and inflammation as central mechanisms [14].

Venous events, although numerically less frequent than arterial ones, showed a higher age-standardized event ratio, underscoring a specific venous vulnerability in EGPA [52]. DVT and pulmonary embolism occurred in 3.3% and 0.9% of patients, respectively, with additional reports of superficial thrombophlebitis, atypical-site thrombosis and intracardiac thrombi [52, 53].

In the Swedish registry-based AAV cohorts, the myocardial infarction incidence rate in EGPA was 0.1 per 100 person-years (95% CI 0.0–3.8) [8]. In the same Swedish study, the overall AAV stroke incidence rate was 11.3 per 1000 person-years (95% CI 6.9–15.8), but an EGPA-specific standardized incidence ratio for stroke was not reported [10]. Likewise, phenotype-specific standardized incidence ratios for DVT and pulmonary embolism were not reported for EGPA [9].

Cardiac involvement is a hallmark of EGPA and represents a major determinant of prognosis. In a monocentric cohort of newly diagnosed patients, cardiac manifestations were identified in 35%, including pericarditis, cardiomyopathy with heart failure, myocarditis and valvular abnormalities [54]. Myocarditis was frequently subclinical and detected by cardiac biomarkers and cardiac MRI. Similar findings were reported in prospective cohorts, with additional observations of apical thrombosis, non-atherosclerotic coronary involvement and apical aneurysms, often associated with higher eosinophil counts at diagnosis [55]. Collectively, these data confirm EGPA as the AAV subtype with the highest burden of direct cardiac involvement [3, 14, 56].

## Assessment of CV disease: biomarkers and imaging modalities

Traditional CV risk scores developed for the general population may underestimate risk in AAV. Tools such as the Framingham risk score do not incorporate inflammatory burden, whereas a disease-specific model has shown improved predictive performance within the first 5 years after diagnosis [11, 57]. In the study by Suppiah *et al.*, the Framingham Risk Score showed an area under the receiver operating characteristic curve (AUROC) of 0.65, indicating a modest CV risk predictive capacity in the AAV cohort, compared with the new model described (AUROC of 0.73 in the original cohort and of 0.80 on the external validation cohort) [57]. This model includes ANCA type, accounting for disease-specific inflammatory burden to estimate CV risk in AAV. Whether recalibration strategies or disease-specific multipliers, as proposed in other immune-mediated diseases such as rheumatoid arthritis, could be applied to AAV remains to be determined. Chronic immune-mediated disorders are recognized as risk modifiers in CV risk assessment [58], and disease activity has independent prognostic relevance. The Birmingham Vasculitis Activity Score (BVAS) has been associated with CV mortality in AAV, supporting the link between inflammatory burden and adverse outcomes [59].

**Figure 2:**
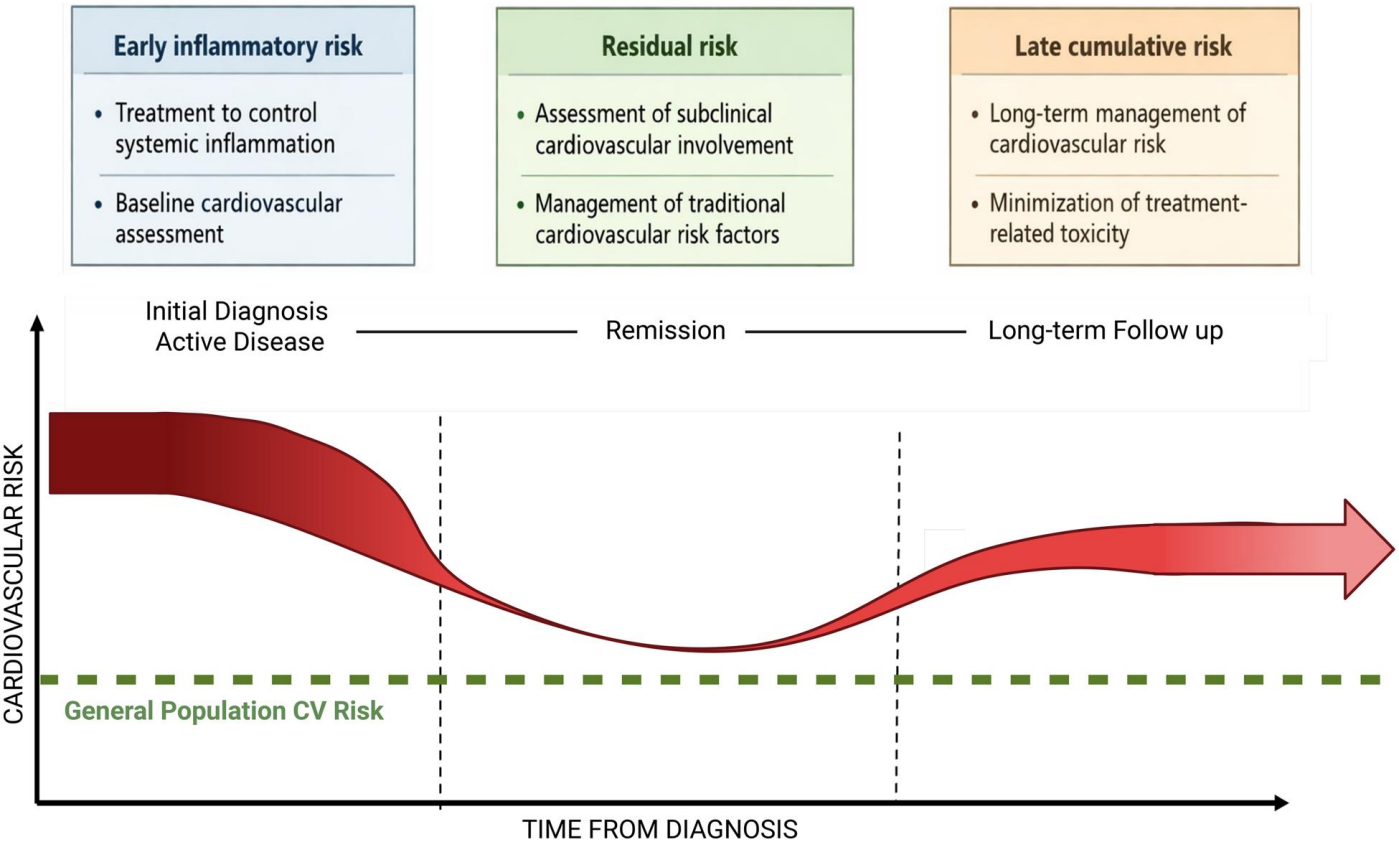
Conceptual model of a bimodal cardiovascular risk profile in AAV. The figure illustrates a time-dependent, conceptual model of cardiovascular (CV) risk in AAV, derived from an overall synthesis of heterogeneous cohorts. CV risk is highest around diagnosis, paralleling peak systemic inflammation (‘early inflammatory risk’). Following remission, risk decreases but remains persistently higher than that of the general population (lower dashed line), reflecting subclinical inflammation and traditional CV risk factors (‘residual risk’). During long-term follow-up, CV risk increases again, although not reaching the initial peak, in relation to cumulative treatment exposure and progressive comorbidity burden (‘late cumulative risk’). This model represents a simplified framework; in clinical practice, individual trajectories are heterogeneous and influenced by relapses, baseline comorbidities and treatment strategies

CV risk in AAV follows a dynamic pattern, with higher event rates observed around diagnosis and during periods of active disease, and persistent excess risk during remission. In addition to inflammatory activity, cumulative treatment exposure, particularly glucocorticoids, and progressive accrual of traditional risk factors likely contribute to long-term risk (Fig. 2).

### Biomarkers

Routine laboratory evaluation primarily reflects systemic inflammation and disease activity. Complete blood count (including eosinophil count in EGPA), CRP, ESR, ANCA titres, urinalysis and proteinuria provide information on inflammatory burden and organ involvement. A higher platelet count at diagnosis has been identified as an independent predictor of stroke risk in AAV [10].

Assessment of traditional CV risk factors remain essential. Fasting glucose, glycated haemoglobin (HbA1c) and a complete lipid profile should be routinely evaluated. Measurement of lipoprotein(a) may refine risk stratification; however, its prognostic significance in AAV remains uncertain despite evidence of elevated levels in other systemic autoimmune diseases. Markers of myocardial injury, including N-terminal pro-B-type natriuretic peptide (NT-proBNP) and high-sensitivity cardiac troponin, may support the identification of overt or subclinical cardiac involvement, particularly in patients with suggestive clinical or imaging findings. Their role in systematic screening has not been formally established.

### Imaging modalities

Imaging contributes to the detection of subclinical vascular damage and cardiac involvement. Several studies have demonstrated increased arterial wall thickness, pulse-wave velocity and carotid intima–media thickness in patients with AAV compared with healthy controls, indicating persistent vascular injury [60]. Carotid ultrasonography to assess plaques and intima–media thickness may therefore assist in CV risk stratification [61].

Cardiac evaluation with electrocardiogram and echocardiography has prognostic relevance. Abnormalities detected by these modalities have been associated with increased all-cause and CV mortality during follow-up [62]. Reported findings include left ventricular hypertrophy, ST-T abnormalities and QTc prolongation on electrocardiogram [33] as well as reduced left ventricular ejection fraction, elevated filling pressures and valvular abnormalities on echocardiography [63]. Cardiac magnetic resonance imaging (CMR) provides additional sensitivity for detecting myocardial inflammation and fibrosis. Late gadolinium enhancement and abnormal native T1 and T2 values may be present even in patients with normal electrocardiogram and echocardiography findings [64]. However, there are currently insufficient data to support the routine use of CMR in asymptomatic patients, although CMR should be considered in the presence of clinical, laboratory or imaging findings suggestive of cardiac involvement [54]. More invasive investigations, including stress echocardiography, coronary angiography and endomyocardial biopsy, should be considered in selected cases based on clinical presentation. In the absence of AAV-specific evidence, coronary artery calcium scoring should be interpreted according to current recommendations for CV risk assessment in the general population [49].

## Morbidity and mortality associated with CV disease

Overall age-adjusted mortality in AAV remains significantly higher than in the general population, ∼1.5-fold (22.5 deaths per 1000 person-years) [65]. CV causes account for nearly 26% of deaths beyond the first year after diagnosis and represent the leading driver of late mortality, whereas infection and active vasculitis predominate in the early disease phase [6, 66]. CV-specific mortality has been estimated to be ∼2.3-fold higher than in the general population [7]. Compared with PR3-ANCA-positive patients, MPO-ANCA-positive patients show a markedly higher risk of CV-related death despite similar all-cause mortality [7]. This difference is likely multifactorial, reflecting differences in age, renal involvement, vascular phenotype and possibly intrinsic pathogenic mechanisms. CV events display a distinct temporal distribution. Incidence is highest within the first months to 2 years after diagnosis, reaching up to 6.89 per 100 person-years (rate ratio 3.43 vs controls) [11]. Coronary ischaemic events have also been reported to occur more frequently within 0–4 years after diagnosis and again after 10 years or more of disease duration [66].

In matched cohort analyses, AAV was associated with a 2.23-fold increased risk of any CV event compared with non-inflammatory chronic kidney disease (CKD). Dialysis dependency, smoking, older age and impaired renal function after remission were independent predictors of future events [67]. Beyond arterial and venous events, direct cardiac involvement represents an important contributor to morbidity. In patients with EGPA and GPA in remission, systematic screening identified cardiac abnormalities in 62% and 46% of patients, respectively, by electrocardiogram and echocardiography, with detection increasing to 66% and 61% when CMR imaging was performed [62]. Imaging-detected abnormalities were associated with increased all-cause and CV mortality during follow-up [62]. EGPA is consistently associated with the highest burden of clinical and subclinical cardiac involvement and with adverse prognosis [34]. Disease-specific factors further influence outcomes. High disease activity at presentation (BVAS), severe renal impairment (eGFR <15 ml/min), dialysis dependency and MPO-ANCA serotype have all been associated with increased CV risk [6, 59, 68, 69].

CV burden in AAV also affects quality of life, contributing to impaired physical and mental health domains and functional limitation [66]. Despite this impact, implementation of preventive strategies remains suboptimal, with inadequate lipid and blood pressure control and progressive accumulation of treatment-related risk factors over time [70, 71].

## Management of CV disease in AAV

No randomized trials have evaluated CV prevention strategies in AAV. Management therefore relies on indirect evidence and must address the multidimensional CV risk arising from inflammation, organ damage, traditional risk factors and treatment-related toxicity.

### Induction and maintenance of remission

Remission induction in AAV is traditionally based on high-dose glucocorticoids combined with either rituximab (RTX) or CYC, followed by maintenance therapy with RTX or conventional DMARDs, particularly in GPA and MPA. While these strategies have markedly improved early survival, glucocorticoids remain a major contributor to hypertension, hyperglycaemia, dyslipidaemia and weight gain, even at relatively low doses [72]. Small mechanistic studies suggest that suppression of inflammation may improve surrogate markers of vascular function. Restoration of endothelial function after CYC therapy has been described in systemic necrotizing vasculitis [73], and inflammation-dependent increases in arterial stiffness have been shown to normalize during remission following immunosuppressive treatment [74]. However, these studies are limited in size and focus on surrogate endpoints, and do not demonstrate reduction in CV events. More recently, glucocorticoid-sparing approaches have been validated for disease control. In PEXIVAS, a reduced-dose glucocorticoid regimen was noninferior to standard dosing for death or end-stage kidney disease in severe AAV [75]. Similar results were observed in LoVAS for reduced-dose glucocorticoids plus RTX in selected newly diagnosed patients [76], and RITAZAREM findings were consistent with this approach [77]. The ADVOCATE trial showed that the C5a receptor inhibitor avacopan, combined with RTX or CYC, was noninferior to prednisone taper for remission at week 26 and superior for sustained remission at week 52 [78], allowing significant reduction in glucocorticoid exposure. However, avacopan availability still varies among different countries, limiting the current overall clinical impact of this corticosteroid-sparing option.

In EGPA, glucocorticoids remain central, but anti-IL-5 therapies (i.e. mepolizumab and benralizumab) have become established maintenance options in relapsing disease, with documented glucocorticoid-sparing effects [79, 80]. In a large Italian EGPA cohort by Bettiol and colleagues, patients not receiving immunomodulating therapy within the first 2 months after diagnosis had a significantly higher risk of acute arterial and venous thromboembolic events compared with those treated with systemic glucocorticoids (HR 3.67, 95% CI 1.37–9.89). In the same study, antiplatelet, anticoagulant or statin therapy at diagnosis was not associated with a reduced risk of thromboembolic events [52].

Taken together, available evidence supports the concept that effective control of inflammatory activity may influence early CV risk, whereas strategies that reduce cumulative glucocorticoid exposure may be relevant to long-term risk mitigation. Direct evidence linking specific immunosuppressive regimens to reduced CV events, however, remains lacking.

### Traditional CV risk factors and residual risk

Even in remission, CV risk in AAV does not normalize. Long-term observational data show progressive accumulation of hypertension and diabetes over time [81], and dyslipidaemia is common, particularly during early disease phases and glucocorticoid exposure [70]. CKD, frequently resulting from prior glomerulonephritis, further amplifies CV risk. Evidence specific to AAV is limited, and management of traditional CV risk factors largely follows recommendations derived from the general population and CKD guidelines. In EUVAS long-term follow-up, hypertension prevalence increased by 36.7% over a mean of 7.1 years [81]. In a German cohort, only 24.3% of AAV patients eligible for statin therapy were receiving treatment [71], highlighting potential undertreatment. Although statins are recommended for high-risk patients in AAV guidelines [2], the STATVAS trial [82] did not demonstrate significant differences in surrogate atherosclerosis markers between rosuvastatin and placebo, despite effective LDL-cholesterol reduction, which does not exclude a potential benefit on CV events, as the trial was not powered for this end point. Observational data in immune-mediated inflammatory diseases suggest possible benefit of statins on CV outcomes, but disease-specific evidence in AAV remains limited [83]. Blood pressure targets and renin–angiotensin system blockade are generally extrapolated from CKD guidance, including KDIGO recommendations, particularly in patients with albuminuria [84]. Similarly, lipid-lowering strategies follow ESC/EAS recommendations for high- and very-high-risk individuals [58]. CKD management in AAV adheres to nephrology guidelines, as established fibrotic damage is not responsive to immunosuppression. Optimization of lifestyle factors (balanced diet, ideal body weight, smoking cessation) is part of general management of CV risk and should not be neglected also in AAV.

Overall, traditional CV risk factor control represents a key component of residual risk management in AAV, although the absence of disease-specific randomized data underscores the need for structured multidisciplinary risk reassessment and individualized therapeutic adjustment over time (Table 2).

**Table 2:** Cardiovascular risk assessment and management in AAV: overview of international guidelines.

Guideline	Risk assessment	Screening	Lipid management	Hypertension	Diabetes
EULAR (2022)	Recommends CV risk assessment beyond traditional models (e.g. SCORE); acknowledges inflammation as CV risk modifier in RMDs	Regular assessment of BP, lipids, BMI, glucose; repeated according to individual risk; encourages multidisciplinary approach	No AAV-specific lipid targets; lipid management according to global CV risk	Tight BP control, particularly with kidney involvement; ACEi/ARB preferred in CKD or proteinuria	Routine screening; diabetes considered major modifiable CV risk factor
ACR/VF (2021)	No dedicated CV risk model for AAV; emphasizes comorbidity assessment and glucocorticoid minimization	Routine CV evaluation encouraged; no structured screening algorithm	No specific lipid recommendations for AAV; follow general CV prevention guidelines	Treat hypertension, especially in renal disease; no defined BP targets	Standard diabetes management; attention to glucocorticoid-induced hyperglycaemia
KDIGO (2021–2024)	Applies traditional CV risk models in CKD populations	Routine BP monitoring; albuminuria and kidney function assessment in CKD	Recommends statin-based therapy in most CKD adults (≥50 years), independent of baseline LDL in many cases	Target SBP <120 mmHg in CKD (standardized measurement); ACEi/ARB preferred in albuminuria	Comprehensive diabetes care in CKD; SGLT2 inhibitors and GLP-1 receptor agonists when appropriate

Cardiovascular prevention in AAV is currently extrapolated from general rheumatologic, nephrologic and cardiovascular recommendations. None of the available guidelines provides AAV-specific cardiovascular risk models, structured screening algorithms or disease-specific therapeutic targets, underscoring a relevant unmet need in this field.

EULAR, European Alliance of Associations for Rheumatology; ACR, American College of Rheumatology; VF, Vasculitis Foundation; KDIGO, Kidney Disease: Improving Global Outcomes; CV, cardiovascular; RMDs, rheumatic and musculoskeletal diseases; SCORE, Systematic COronary Risk Evaluation; BP, blood pressure; AAV, ANCA-associated vasculitis; ACEi, angiotensin-converting enzyme inhibitor; ARB, angiotensin receptor blocker; CKD, chronic kidney disease; SBP, systolic blood pressure; LDL, low-density lipoprotein; SGLT2 inhibitors, sodium-glucose cotransporter-2 inhibitors; GLP-1 receptor agonists, glucagon-like peptide-1 receptor agonists.

## Unmet needs and future directions

Over the past decade, therapeutic strategies in AAV have substantially improved, particularly regarding remission induction and reduction of cumulative glucocorticoid exposure. Nevertheless, CV disease remains a major determinant of long-term morbidity and mortality. While early inflammatory burden contributes to vascular events around diagnosis and during relapses, excess risk persists during clinical remission.

Several unmet needs remain. First, the clustering of CV events within the first months after diagnosis suggests that inflammatory control alone may be insufficient to mitigate early thrombotic risk. Whether adjunctive short-term antithrombotic strategies during this high-risk phase improve outcomes have not been evaluated. Second, no prospective trials have incorporated CV outcomes as predefined endpoints in AAV; the impact of different immunosuppressive regimens is therefore largely inferred from surrogate markers or non-controlled studies. Third, optimal CV risk stratification in AAV remains undefined. Traditional risk scores may underestimate risk, and validated disease-specific algorithms are lacking. Fourth, although subclinical vascular and cardiac abnormalities are frequently detected by imaging, their integration into routine clinical decision-making is not standardized.

Management of traditional CV risk factors is largely extrapolated from general population guidelines. Whether intensified lipid-lowering strategies or stricter blood pressure targets improve long-term outcomes in AAV remains uncertain. Similarly, the role of SGLT2 inhibitors in AAV remains undefined, as patients with active autoimmune kidney disease were excluded from major randomized trials. Likewise, the role of biomarkers reflecting persistent low-grade inflammation in guiding preventive strategies requires clarification.

Future research should integrate CV endpoints into AAV-specific studies and refine risk stratification across disease phases. Until such data become available, CV management in AAV relies on strict disease control, minimization of glucocorticoid exposure, systematic multidisciplinary assessment of traditional risk factors and individualized clinical judgement.

## Data Availability

There are no new data associated with this article.
